# Colleges in the United States

**Published:** 1850-03

**Authors:** 


### COLLEGES IN THE UNITED STATES.

There are, in the United States, one hundred and twenty colleges, of which eighty-one have been chartered since 1819. Many of them, in fact, have only a nominal existence. The oldest are, Harvard College, in Massachusetts, founded in 1636; William and Mary, in Virginia, in 1692; Yale College, New Haven, Conn., in 1700; Princeton, in New

Jersey, in 1746, and the University of Pennsylvania, in 1755. The several religious persuasions are very variously represented, as respects the numbers of the literary institutions attached to their several interests. Thus, seventy-one, (or considerably more than half,) are representatives of the Presbyterian and Congregational denominations, or are undesignated, and this number embraces by far the largest and most celebrated of American literary institutions—e. g., Yale, Harvard, Princeton, &c. The Baptists, Romanists, and Methodists have each thirteen, and the Episcopalians ten colleges. The literary property of these several institutions, is as various as their other points of comparison. It appears that the aggregate libraries of the American colleges is 751,425 volumes. The several libraries at Harvard contain nearly 85,000 volumes which is the largest collection of books in America. The next largest collection of books is found at Yale, where the number is nearly 50,000. Below these we find Brown University, Bowdoin, and Georgetown, having respectively 27,520, 25,520, and 23,250 volumes each.—Harvard College has 6,203 graduates, of whom 1,628 have been clergymen. Yale has had 5,856 graduates, of whom 1,510 have been clergymen. Princeton has the next largest number of graduates, 3,031, of whom 577 have been clergymen. If the medical graduates of Pennsylvania University are included in her list of names, the aggregate graduated at that institution rises to 5,142, of which, however, 4,952 are medical. It is difficult to form a just estimate of the corps of officers engaged in the several institutions, since in the Roman Catholic colleges (?) the teachers in the primary departments are included, as well as the scolars in the primary departments. Bearing this in mind, we find only five American colleges, in which over twenty instructors are employed, viz: Mt. St. Mary's, Baltimore, (Catholic primary and collegiate) twenty-four teachers. Yale, twenty-one. St. Charles, Louisiana, (Catholic), twenty-one. Harvard, twenty. St. Mary's, twenty.

The colleges are very unequally distributed over the union. The older States, with a dense population, numerous schools, and academies, and minor means of instruction have, as a general rule, fewest colleges; but those they have, are far more numerously attended, and better endowed, than the more frequent but often feeble institutions in the South and West. Thus, Maine has two colleges; New Hampshire, one; Massachusetts, three; Vermont, three; Rhode Island, one; Connecticut, three; New York, eight; New Jersey, three; Pennsylvania, ten; Delaware, one; Maryland, five; District of Columbia, two; Virginia, ten; North Carolina, t ree; South Carolina, three; Georgia, five; Alabama, four; Mississippi, one; Louisiana, four; Tennessee, eight; Kentucky, eight; Ohio, twelve; Indiana, six; Illinois, four; Missouri, six; Michigan, two; Wisconsin, one.

In all this long list, the only literary institution whose aggregate number of students in the *Academical* department, (not preparatory) exceeds three hundred students, is Yale College, in Connecticut. This institution has 306 students this year, and last year (1848-49) had 385 academical students. Seven other institutions exceed two hundred students, and twenty-nine have between one and two hundred. The annual expense of tuition, at these institutions, ranges from $24 to $75—a very moderate charge, certainly, at the highest, for the time and services of so many competent instructors.

*Medical Schools*—At the last dates there were thirty-five medical schools in the United States, viz: in Pennsylvania, five; in New York, four; in Maryland, two; in Massachusetts, two; in Vermont, two; in Virginia, two; in Kentucky, two; in Ohio, three; and in Missouri, four. In the other States, only one exists, or none, in each State. The oldest medical school in the United States is the old medical department of the University of Pennsylvania, which was established in 1765. Next, we have the medical school of Harvard College, founded in 1782, and that of Dartmouth, in New Hampshire, established in 1797.

The oldest institution in the West, for medical instruction, is at Lexington, founded in 1818, and having now 1,351 graduates. The numbers of graduates at Pennsylvan a University is 4,952; at Jefferson Medical College, 1,598; at Baltimore, 909; at the College of Physicians and Surgeons, New York, 852; at Yale, 850; at Louisville, 688; at the University of New York, 597; New Hampshire, 758; Maine medical school, 596, &c. The institutions having the largest classes at present, are the University of Pennsylvania, 508; Jefferson Medical College, 480; Medical Faculty at the New York University, 421; Louisville, 376; Cleveland, 258; the College of Physicians and Surgeons, New York, 219. The number of Professors in the several schools ranges from three to nine, viz: one has three, (University of Virginia); three have five; thirteen have six; thirteen have seven; three have eight; two have nine, (the St. Louis colleges.)

The growth of the medical department of the Louisville University has been remarkably regular and progressive. We annex the statistics in a tabular form, viz:

| *Years.* | *Students.* | *Graduates.* | *Years.* | *Students.* | *Graduates.* |
|---|---|---|---|---|---|
| 1837-38 | 80 | 24 | 1844-45 | 286 | 71 |
| 1838-39 | 120 | 27 | 1845-46 | 345 | 73 |
| 1839-40 | 204 | 38 | 1846-47 | 348 | 75 |
| 1840-41 | 208 | 48 | 1847-48 | 406 | 94 |
| 1841-42 | 262 | 53 | 1848-49 | 332 | 81 |
| 1842-43 | 189 | 37 | 1849-50 | 376 | |
| 1843-44 | 242 | 47 | | | |

